# Does the Interaction Between Cortisol and Testosterone Predict Men’s Facial Attractiveness?

**DOI:** 10.1007/s40750-017-0064-1

**Published:** 2017-03-30

**Authors:** Michal Kandrik, Amanda C. Hahn, Chengyang Han, Joanna Wincenciak, Claire I. Fisher, Lisa M. DeBruine, Benedict C. Jones

**Affiliations:** 1 Institute of Neuroscience and Psychology, College of Medical, Veterinary and Life Sciences, University of Glasgow, Glasgow, G12 8QB UK; 2 Present Address: Department of Psychology, Humboldt State University, Arcata, CA USA; 3 Present Address: Institute of Neuroscience, Newcastle University, Newcastle, UK

**Keywords:** Attractiveness, Dominance, Health, Faces, Testosterone, Cortisol

## Abstract

Although some researchers have suggested that the interaction between cortisol and testosterone predicts ratings of men’s facial attractiveness, evidence for this pattern of results is equivocal. Consequently, the current study tested for a correlation between men’s facial attractiveness and the interaction between their cortisol and testosterone levels. We also tested for corresponding relationships between the interaction between cortisol and testosterone and ratings of men’s facial health and dominance (perceived traits that are correlated with facial attractiveness in men). We found no evidence that ratings of either facial attractiveness or health were correlated with the interaction between cortisol and testosterone. Some analyses suggested that the interaction between cortisol and testosterone levels may predict ratings of men’s facial dominance, however, with testosterone being more closely related to facial dominance ratings among men with higher cortisol. Our results suggest that the relationship between men’s facial attractiveness and the interaction between cortisol and testosterone is not robust.

**Electronic supplementary material:**

The online version of this article (doi:10.1007/s40750-017-0064-1) contains supplementary material, which is available to authorized users.

## Introduction

Male secondary sexual characteristics are dependent on testosterone levels in multiple species (Andersson 1994). Experimentally increasing testosterone levels impairs immune function in males (see Foo et al. 2016 for a meta-analytic review). Since it has been suggested that only males in good physical condition will be able to maintain healthy development in the face of this testosterone-linked immunosuppression (Folstad and Karter 1992) men with high testosterone levels may appear to be attractive, healthy, and dominant (Penton-Voak and Chen 2004; Roney et al. 2006). Given the importance of facial cues for human social interactions (see Little et al. 2011 for a review), much of the work testing these predictions has focused on possible links between testosterone and aspects of men’s facial appearance.

Early research investigating possible links between testosterone and perceptions of men’s facial appearance reported that the faces of men with higher basal testosterone levels were perceived to be more masculine (Penton-Voak and Chen 2004; Roney et al. 2006) and more attractive as short-term partners (Roney et al. 2006). However, other studies did not observe significant associations between men’s basal testosterone and their facial attractiveness, dominance or masculinity (Hönekopp et al. 2007; Neave et al. 2003; Pound et al. 2009). Similarly, studies investigating possible relationships between aspects of facial morphology, such as facial width-to height ratio, and men’s testosterone levels have also observed little evidence for correlations between men’s facial appearance and testosterone levels (e.g., Whitehouse et al. 2015; for a meta-analytic review see Bird et al. 2016).

Although short-term increases in cortisol levels stimulate immune responses, chronically elevated cortisol levels are associated with immunosuppression (Sapolsky et al. 2000; Martin 2009). Consequently, some researchers have suggested that the inconsistent results described above could be a consequence of studies having not considered the possible moderating role of cortisol on the association between testosterone and men’s facial appearance (Rantala et al. 2012; Moore et al. 2011a, 2011b). Such research has also produced mixed results, however. Rantala et al. (2012) observed a significant interaction between the effects of testosterone and cortisol for men’s facial attractiveness, whereby the positive correlation between testosterone and attractiveness was stronger among men with low cortisol than it was among men with high cortisol. However, Moore et al. (2011a, 2011b) did not replicate these findings and also found no evidence that cortisol moderated the relationship between testosterone and dominance or health ratings of men’s faces.^1^

In light of the inconsistent results described in the previous paragraph, we tested whether cortisol moderated the relationship between salivary testosterone and ratings of men’s facial attractiveness, health, and dominance.

## Methods

### Participants

Forty-five heterosexual men participated in the study (mean age = 21.97 years, SD = 3.31 years). All participants were students at the University of Glasgow (Scotland, UK). None of these men were currently taking any form of hormonal supplement and all indicated that they had not taken any form of hormonal supplement in the 90 days prior to participation. One additional man was tested but excluded from the dataset because his average cortisol level was more than five standard deviations above the mean for the rest of the sample. Forty-three men reported white ethnicity, 1 man reported Arabic ethnicity, and 1 man did not report his ethnicity.

### Procedure

All participants completed five weekly test sessions. All test sessions took place between 2 pm and 5 pm to minimize diurnal variation in hormone levels (Papacosta and Nassis 2011). During each test session, participants provided a saliva sample via passive drool (Papacosta and Nassis 2011). Participants were instructed to avoid consuming alcohol and coffee in the 12 h prior to participation and avoid eating, smoking, drinking, chewing gum, or brushing their teeth in the 60 min prior to participation. Saliva samples were frozen immediately and stored at −32 °C until being shipped, on dry ice, to the Salimetrics Lab (Suffolk, UK) for analysis, where they were assayed using the Salivary Testosterone Enzyme Immunoassay Kit 1–2402 (M = 182.10 pg/mL, SD = 43.15 pg/mL, intra-assay CV = 4.6%, inter-assay CV = 9.83%, sensitivity = < 1.0 pg/mL) and the Salivary Cortisol Enzyme Immunoassay Kit 1–3002 (M = 0.19 μg/dL, SD = 0.07 μg/dL, intra-assay CV = 3.5%, inter-assay CV = 5.1%, sensitivity = < 0.003 μg/dL). All assays passed Salimetrics’ quality control. The average intra-class correlation for testosterone was .92 and for cortisol .73 suggesting good consistency across the 5 test sessions for both hormones.

In each of the five test sessions, each participant first cleaned his face with hypoallergenic face wipes. A full-face digital photograph was taken a minimum of 10 min later. Photographs were taken in a small windowless room against a constant background, under standardized diffuse lighting conditions, and participants were instructed to pose with a neutral expression. Camera-to-head distance and camera settings were held constant. Participants wore a white smock covering their clothing when photographed. Photographs were taken using a Nikon D300S digital camera and a GretagMacbeth 24-square ColorChecker chart was included in each image for use in color calibration. Following other recent work on social judgments of faces (e.g., Jones et al. 2015), face images were color calibrated using a least-squares transform from an 11-expression polynomial expansion developed to standardize color information across images (Hong et al. 2001). Images were masked so that hairstyle and clothing were not visible and standardized on pupil positions.

Next, the face photographs of the 45 men (225 face photographs in total, five face photographs of each man) were rated for attractiveness, health, and dominance using 1 (low) to 7 (high) scales. Attractiveness, health and dominance were each rated in separate blocks of trials. Trial order was fully randomized within each block of trials. Thirty men and 43 women (mean age = 23.22 years, SD = 4.27 years) rated the faces with each individual rater randomly allocated to rate between 2 and 4 blocks of trials (mean number of raters per block of trials = 32.3, SD = 2.89). All raters were students at the University of Glasgow (Scotland, UK). Fifty-six raters reported white ethnicity, 5 reported mixed ethnicity, 4 reported East Asian ethnicity, 3 reported West Asian ethnicity, 1 reported Arabic ethnicity, and 4 reported other ethnicity. One rater chose not to report their age. Inter-rater agreement was high for each trait (all Cronbach’s alphas > .94). Men’s and women’s ratings were also strongly positively correlated for all traits (all *r* > .89). Consequently, we calculated the mean dominance (M = 3.59, SD = 0.75), attractiveness (M = 2.89, SD = 0.59), and health (M = 3.97, SD = 0.60) rating for each man’s face. Mean attractiveness, health, and dominance ratings were positively intercorrelated (all *r* > .60). The average intra-class correlation for each trait was >.90.

## Results

Because cortisol was significantly skewed, natural log-transformed values were used in the analyses (following Mehta and Josephs 2010). First, we investigated the variation in ratings of men’s faces using regression analyses, in which average testosterone level (centered on the group mean and scaled), natural log-transformed cortisol level (centered on the group mean and scaled), and the interaction term were entered simultaneously as predictors. For attractiveness, the analysis revealed no significant effects of testosterone (standardized beta =0.03, *t* = 0.13, *p* = .896, 95% CI: –0.39, 0.44), cortisol (standardized beta = − 0.19, *t* = − 0.97, *p* = .337, 95% CI: –0.58, 0.20), or their interaction (standardized beta = − 0.005, *t* = − 0.04, *p* = .970, 95% CI: –0.27, 0.26). The analyses of health and dominance ratings also revealed no significant effects of testosterone (health: standardized beta = −0.10, *t* = − 0.51, *p* = .612, 95% CI: –0.52, 0.31; dominance: standardized beta =0.12, *t* = 0.61, *p* = .545, 95% CI: –0.28, 0.52), cortisol (health: standardized beta = −0.14, *t* = −0.72, *p* = .478, 95% CI: –0.53, 0.25; dominance: standardized beta = − 0.07, *t* = − 0.38, *p* = .709, 95% CI: –0.45, 0.31), or their interaction (health: standardized beta =0.08, *t* = 0.59, *p* = .560, 95% CI: –0.19, 0.35; dominance: standardized beta =0.23, *t* = 1.78, *p* = .083, 95% CI: –0.03, 0.49). Repeating these analyses controlling for participant age did not alter the pattern of results.

**Fig. 1:**
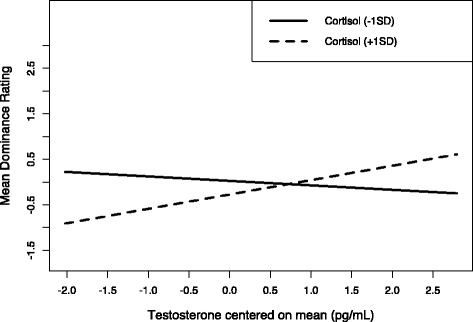
The interaction between average testosterone and average cortisol for men’s dominance. There was a positive relationship between dominance ratings and testosterone at 1SD above average cortisol, although this relationship was not significant (simple slope = 0.35, *t* = 1.77, *p* = .084). At 1SD below average cortisol, however, the slope was negative (simple slope = −0.11, *t* = −0.40, *p* = .692)

Next, following Rantala et al. (2012), we conducted separate bivariate analyses examining the effects of testosterone, cortisol, and their interaction individually. For attractiveness, there was no significant effect of testosterone (F = 0.35, df = 1, 43, standardized beta = −0.09, *p* = .555, adjusted R^2^ = −.015, 95% CI: –0.40, 0.22), cortisol (F = 1.33, df = 1, 43, standardized beta = −0.17, *p* = .255, adjusted R^2^ = .007, 95% CI: –0.48, 0.13), or their interaction (F = 0.07, df = 1, 43, standardized beta = −0.03, *p* = .795, adjusted R^2^ = −.022, 95% CI: –0.28, 0.21). For health ratings, there was no significant effect of testosterone (F = 0.98, df = 1, 43, standardized beta = −0.15, *p* = .327, adjusted R^2^ = −.0003, 95% CI: –0.45, 0.15), cortisol (F = 1.42, df = 1, 43, standardized beta = − 0.18, *p* = .240, adjusted R^2^ = .009, 95% CI: –0.48, 0.12), or their interaction (F = 0.02, df = 1, 43, standardized beta =0.02, *p* = .876, adjusted R^2^ = −.023, 95% CI: –0.23, 0.26). For dominance ratings, there was no significant effect of testosterone (F = 1.63, df = 1, 43, standardized beta = −0.191, *p* = .208, adjusted R^2^ = .014, 95% CI: –0.11, 0.49) or cortisol (F = 0.21, df = 1, 43, standardized beta = −0.07, *p* = .645, adjusted R^2^ = − .018, 95% CI: –0.24, 0.38), but their interaction was significant (F = 4.80, df = 1, 43, standardized beta =0.25, *p* = .034, adjusted R^2^ = .08, 95% CI: 0.02, 0.48). To interpret this two-way interaction we carried out a simple slopes analysis. This analysis showed that there was a positive relationship between dominance ratings and testosterone at 1SD above average cortisol, although this relationship was not significant (simple slope = 0.35, *t* = 1.77, *p* = .084). At 1SD below average cortisol, however, the slope was negative (simple slope = −0.11, *t* = −0.40, *p* = .692) (Fig. 1).

## Discussion

Here we investigated whether cortisol moderated the relationship between testosterone and men’s facial attractiveness. By contrast with Rantala et al. (2012), who found that testosterone levels were more positively related to facial attractiveness in men with low cortisol levels than in men with high cortisol levels, we found no evidence that the interaction between testosterone and cortisol was related to men’s facial attractiveness. Although Rantala et al. (2012) had a slightly larger sample size than we did (*N* = 62 in their study, versus *N* = 45 in our study) lower power is unlikely to explain this discrepancy in results; while Rantala et al. (2012) reported standardized beta of .26 for the effect of the interaction between testosterone and cortisol on attractiveness in their study, the corresponding standardized beta in our study was −.03. Our null results for the interaction between testosterone and cortisol for attractiveness ratings are also consistent with null results reported by Moore et al. (2011a, 2011b). Together, these null results call into question the extent to which cortisol moderates a relationship between facial attractiveness and men’s testosterone levels. Like Moore et al. (2011a), we also saw no evidence that cortisol moderated a relationship between testosterone and ratings of men’s facial health.

Although we saw no evidence that the interaction between testosterone and cortisol predicted men’s facial attractiveness or health, we saw some evidence that the interaction between testosterone and cortisol may be weakly related to ratings of men’s facial dominance. The bivariate relationship between the interaction term and ratings of men’s facial dominance was significant (although the interaction term had no significant effects in the full model, which also included testosterone and cortisol) and simple slope analyses suggested that testosterone was positively related to dominance ratings at 1SD above the mean for cortisol, but not 1SD below the mean for cortisol. Only one other study has tested whether cortisol moderates the relationship between testosterone and facial dominance in men. Consistent with our results, Moore et al. (2011b) found that a prototype face with the average shape, color and texture information of men with high cortisol and low testosterone levels was judged to be less dominant than prototype faces representing men with low cortisol and low testosterone, high cortisol and high testosterone, or low cortisol and high testosterone levels. Moore et al. (2011b) did not examine dominance ratings of individual faces, however. That men with the combination of high testosterone and high cortisol *look* particularly dominant would be consistent with research suggesting that people with this particular hormonal profile actually *are* particularly dominant and/or aggressive (Denson et al. 2013; Welker et al. 2014). However, other studies have found that men with high testosterone and low cortisol tend to be particularly dominant (Mehta and Josephs 2010). Importantly, we emphasize here that the effect of the interaction term for facial dominance is only significant in one of our analyses and even then would not be significant if we corrected for multiple comparisons. This raises the possibility that it is a false positive.

Like previous studies (e.g., Hönekopp et al. 2007; Neave et al. 2003; Moore et al. 2011a, 2011b), we did not observe significant relationships between men’s testosterone or cortisol levels and ratings of their facial attractiveness, health, or dominance. Given our small sample size, and the small sample sizes typical of work on this topic, we cannot rule out the possibility that these null results are false negatives, however. Further research employing larger samples may be necessary to clarify the nature of the relationships between men’s hormone levels and perceived facial appearance.

Rantala et al. (2012) found that cortisol moderated the relationship between men’s facial attractiveness and testosterone levels, such that the relationship between men’s testosterone levels and facial attractiveness was stronger in men with low cortisol levels. This result suggested that inconsistent findings for the relationship between facial attractiveness and testosterone in men might reflect studies failing to consider the effects of cortisol. In our study, however, we find no evidence that cortisol moderates the relationship between testosterone and facial attractiveness in men. Moreover, that the direction of the relationship between facial attractiveness and the interaction term for the effects of testosterone and cortisol in our study was in the opposite direction to that reported by Rantala et al. (2012) suggests our failure to replicate Rantala et al.’s results is not due to our study being underpowered.

## Electronic supplementary material

ESM 1 (XLSX 53 kb)

## Footnotes

1 The two studies by Moore et al. (2011a, 2011b) each tested for associations between hormone levels and facial appearance using both ratings of individual faces and ratings of prototypes manufactured to possess the average shape, color, and texture information of samples of men with different combinations of salivary cortisol and testosterone levels. Because their results for ratings of individual faces speak directly to the question of whether individual faces contain cues to hormone levels, we only discuss Moore et al.’s results for analyses of individual faces here.
